# Avian communities of betel nut agroforestry and secondary forest in Taiwan

**DOI:** 10.3897/BDJ.13.e147871

**Published:** 2025-05-05

**Authors:** Jo-Szu Tsai, Chia-Hao Chang, Ping Huang, Jerome Chie-Jen Ko, Fu-Hsiung Hsu

**Affiliations:** 1 National Chiayi University, Chiayi City, Taiwan National Chiayi University Chiayi City Taiwan; 2 Taiwan Biodiversity Research Institute, Chichi township, Taiwan Taiwan Biodiversity Research Institute Chichi township Taiwan

**Keywords:** avian communities, agroforestry, betel nut, secondary forest, subtropical Asia

## Abstract

**Background:**

Betel nut agroforestry had quickly grown to occupy more than 1% of the land in Taiwan, often taking the place of secondary forests. While agroforestry systems can help mitigate biodiversity loss by preserving habitat structure and providing essential ecological services, the ecological role of betel nut plantations — particularly their impact on avian communities — remains largely understudied. Birds, as sensitive indicators of environmental change, offer valuable insights into habitat quality and biodiversity health. To address this knowledge gap, we conducted standardised point count surveys to collect data on bird species composition and abundance.

**New information:**

This was the first avian dataset focused on betel nut agroforestry, providing detailed bird occurrence data for betel nut plantations and secondary forests. The dataset is particularly useful for understanding how agriculture alters ecological services in human-modified landscapes. Data were collected during the breeding seasons (March to May) of 2015 and 2016 across 80 sites in four counties of central Taiwan. Each site included five sampling points, with avian community data recorded twice per season at each point. This publicly available dataset on GBIF offers valuable insights into avian biodiversity and habitat use in agricultural ecosystems.

## Introduction

The conversion of natural habitats to farmlands drives biodiversity loss globally (Altieri 1999, Dudley and Alexander 2017, Williams et al. 2020), with intensive agriculture further threatening species diversity (Hazell and Wood 2008, Raven and Wagner 2021). Agroforestry systems, such as coffee, oil palm and rubber, can mitigate these impacts by creating refuge habitats, but their biodiversity value depends on management practices (Dietsch et al. 2007, Jose 2009, Karp et al. 2013). As a relatively unexamined system, betel nut agroforestry presents a valuable chance to study biodiversity within agricultural landscapes. Although it maintains tree cover and sustains livelihoods, its ecological advantages and challenges differ. Betel nut agroforestry predominantly exists in the tropical and subtropical zones of South and Southeast Asia, where it plays a vital role in both cultural traditions and economic functions (Pratt 2014, Singh et al. 2020). Despite the association of health concerns and social challenges, betel nut agroforestry plays a crucial economic and cultural role in these regions (Tham et al. 2017, Luong et al. 2024).

In Taiwan, betel nut plantations are a vital type of agroforestry, particularly in the hillside areas of central and southern Taiwan below 1200 m above sea level. The planting area has been increasing forty-fold since the 1960s and peaked in the 1990s due to its high economic value (Liu 2010), exceeding one percent of the total land area in Taiwan. The expansion of betel nut replaced vast areas of secondary forests, raising concerns about their potential impacts on ecological services, particularly in mountainous regions. Betel nut agroforestry systems retain tree cover, but often cause higher runoff and erosion than secondary forests, posing soil and water conservation challenges (Lu et al. 1999, Cheng et al. 2008). Despite its widespread economic significance, the effect of betel nut plantation expansion on biodiversity remains underexplored. Birds are sensitive to environmental change and habitat diversity and are regarded as significant indicators for biodiversity health (Fraixedas et al. 2020). Studying how these mobile organisms engage with betel nut agroforestry will clarify the implications of these plantations on avian community composition and dynamics, especially when compared to secondary forests, which are considered vital biodiversity reservoirs in human-altered landscapes.

The primary aim of this dataset was to address the knowledge gap concerning the ecological impact of betel nut agroforestry on avian biodiversity in central Taiwan. Specifically, we collected the composition and diversity of bird communities in betel nut agroforestry systems with those in adjacent secondary forests. With this dataset, we aimed to provide insights into the potential role of betel nut plantations in supporting avian populations. Such information is essential for guiding sustainable management practices and balancing economic and ecological interests in the region.

## Project description

### Title

Avian communities of betel nut agroforestry and secondary forest in Taiwan

### Study area description

The study area is in the subtropical climate. The annual average temperature is 23℃ and the annual rainfall is 1774.3 mm. The rainy season is from June to August caused by the southwest monsoon.

## Sampling methods

### Sampling description

**Figure 1.**
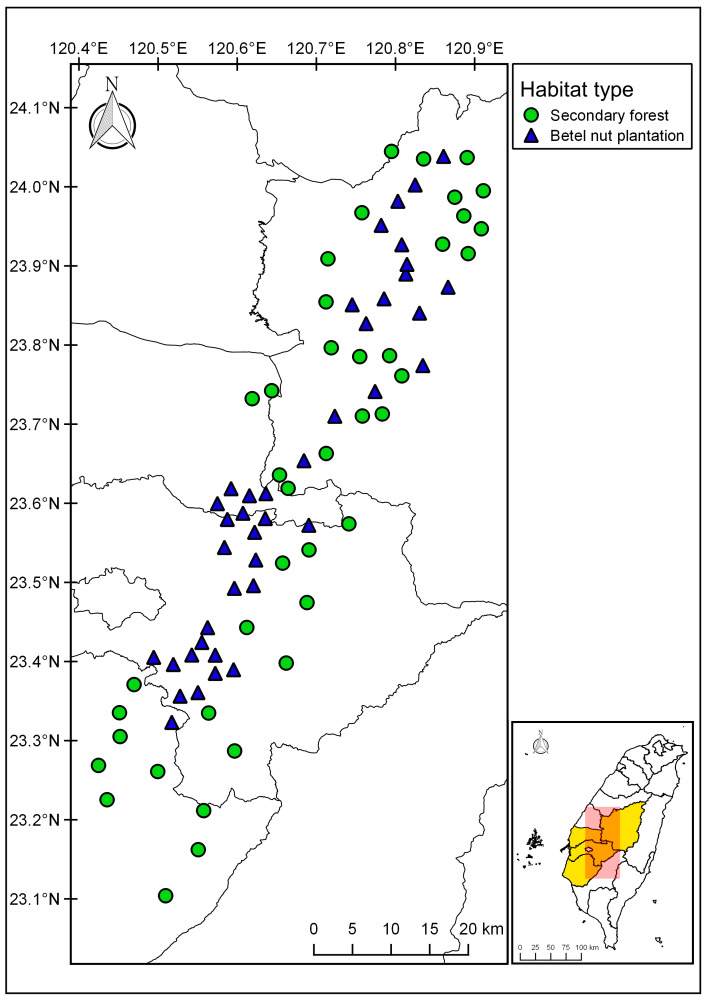
Locations of 40 betel nut agroforestry and 40 secondary forest plots in central Taiwan.

We conducted five point count surveys in 40 betel nut and 40 secondary forest plots in March to May 2015 and 2016 in central Taiwan (Fig. 1). Following a standard point count procedure (Buckland et al. 2005), we spent 6 minutes at each point to record the number of different bird species we saw and heard and the distance of each bird determined by Laser range finder (Nikon laser 1000AS).

We identified homogeneous patches of secondary forest and betel nut plantation below 1200 m above sea level using Google Earth software (Google Inc.) that are larger than 13.6 ha. We then selected 40 secondary forest and 40 betel nut plantation plots that are at least 4 km apart from the plot centre. Within each plot, we established five sampling points along the designated road system, with each point at least 200 m apart to prevent double-counting. Bird sampling was conducted within 4 hours after sunrise on a clear day (without rain and strong wind). Each sampling point was surveyed twice in the breeding season (March to May). For each bird record, the following information was collected: bird species (the smallest taxonomic unit: species), number of individuals (number), horizontal distance to the observer and flock formation with more than five individuals were recorded. In 2015, the horizontal distance was recorded using four categories (0-25 m, 25-100 m, >100 m and flyovers), following the standardised method used in the Taiwan Breeding Bird Survey (Ko et al. 2017). However, we recognised that these broad categories might be too coarse for accurately estimating detection probabilities in future analyses. Therefore, starting in 2016, distance estimation was refined to 10-m bands (in addition to a flyover category) to enhance precision and allow greater flexibility in future data applications. For each sampling point record, investigation date (year, month and day), starting time, observer and weather condition (clear, cloudy, overcast, fog, drizzle or showers) were recorded. Wind conditions during the survey were also recorded and categorised into four levels, based on the Beaufort scale: (1) Calm to Light Air (Beaufort 0–1), (2) Light to Gentle Breeze (2–3), (3) Moderate to Fresh Breeze (4–5) and (4) Strong Breeze and Above (≥ 6). To ensure data collection consistency, the investigation was conducted by four experienced investigators, all of whom had prior field experience in forest bird surveys. At the beginning of the study, all investigators underwent briefings in the field to align and standardise procedures for species identification and distance estimation. A laser rangefinder was used to obtain actual distance measurements, standardising the investigators’ reference points. The taxonomic system follows the Checklist of Birds of Taiwan, maintained by the Bird Record Committee of the Taiwan Wild Bird Federation (Ding et al. 2023).

## Geographic coverage

### Description

The records in this dataset are collected from the central region of Taiwan island, including Nantou, Yunlin, Chiayi and Tainan Counties.

### Coordinates

23.101 and 24.047 Latitude; 120.355 and 120.914 Longitude.

## Taxonomic coverage

### Description

This dataset focused on the avian communities in Taiwan. We recorded 85 species distributed in 11 orders and 39 families. Detailed information of bird order, family and occurrences were described in Table 1.

## Temporal coverage

### Notes

2015-03-01 through 2015-05-31, 2016-03-01 through 2016-05-31.

## Usage licence

### Usage licence

Creative Commons Public Domain Waiver (CC-Zero)

### IP rights notes

This work is licensed under a Creative Commons Attribution (CC-BY 4.0) License.

## Data resources

### Data package title

Avian communities of betel nut plantation and secondary forest in Taiwan

### Resource link

https://doi.org/10.15468/wkdnqx

### Alternative identifiers

6ef6360c-c904-4eab-87fe-7bd234cb5c42

### Number of data sets

1

### Data set 1.

#### Data set name

Avian communities of betel nut plantation and secondary forest in Taiwan.

#### Data format

Darwin Core

#### Description

This was a project focusing on understanding avian communities in the betel nut plantation and the secondary forest in Taiwan. We collected the data in the breeding season (March to May) in 2015 and 2016 in a total of 80 sites in four counties in central Taiwan (Tsai et al. (2025)). There were five sampling points in each site and we collected avian community data at each point twice in each season.

**Data set 1.** 

Column label	Column description
eventID	The identifier for each sampling event.
parentEventID	The identifier for the sampling year (and locationID) for the nested sampling events, each with its own evnetID.
eventDate	The date during which a sampling event occurred.
eventTime	The time during which a sampling event occurred.
samplingProtocol	Protocol used in sampling events.
sampleSizeValue	The area where a sampling event occurred.
sampleSizeUnit	The unit of the area where a sampling event occurred.
samplingEffort	The amount of time spent during a sampling event.
locationID	The identifier for each sampling site.
country	The name of the country where the sampling events occurred.
countryCode	The standard code for the country where the sampling events occurred.
decimalLatitude	The geographic latitude in decimal degrees of the centre of a sampling site.
decimalLongitude	The spatial reference system on which the geographic coordinates of sampling sites were based.
coordinateUncertaintyInMetres	The horizontal distance (in metres) from the given sampling site coordinates describing the smallest circle containing the whole of the sampling site.
Type	The nature of the record resource.
basisOfRecord	The specific nature of the data record.
occurrenceID	The identifier for each occurrence record.
recordedBy	The names of people responsible for each occurrence record.
individualCount	The number of individuals of the species observed during the 6-min observation time.
occurrenceRemarks	Comments or notes about the given occurrence record.
scientificName	The scientific name for the species presented in the occurrence record.
kingdom	The scientific name of the kingdom in which the species is classified.
phylum	The scientific name of the phylum in which the species is classified.
class	The scientific name of the class in which the species is classified.
Order	The scientific name of the order in which the species is classified.
family	The scientific name of the family in which the species is classified.
genus	The scientific name of the genus in which the species is classified.
taxonRank	The taxonomic rank of the most specific name of the species.
vernacularName	Chinese common name for the species.
measurementID	The identifier for each measurement of fact took during sampling.
measurementType	The nature of each measurement.
measurementValue	The value of each measurement.
measurementDeterminedBy	The names of people who took the measurements.
measurementDeterminedDate	The date on which the measurement was made.
measurementMethod	The method or protocol used to determine the measurement.
geodeticDatum	The spatial reference system on which the geographic coordinates of sampling sites were based.

## Figures and Tables

**Table 1.** Taxonomic composition of bird species observed in betel nut agroforestry and secondary forest in Taiwan.

Rank	Order	Family	Scientific name	Occurrences in betel nut agroforestry	Occurrences in secondary forest
1	Accipitriformes	Accipitridae	*Accipitertrivirgatus* (Temminck, 1824)	17	11
2	Accipitriformes	Accipitridae	*Accipitervirgatus* (Temminck, 1822)	2	2
3	Accipitriformes	Accipitridae	*Accipitersoloensis* (Horsfield, 1821)	1	1
4	Accipitriformes	Accipitridae	*Butasturindicus* (Gmelin, 1788)	0	3
5	Accipitriformes	Accipitridae	*Ictinaetusmalayensis* (Temminck, 1822)	1	0
6	Accipitriformes	Accipitridae	*Pernisptilorhynchus* (Temminck, 1821)	2	2
7	Accipitriformes	Accipitridae	*Spilornischeela* (Latham, 1790)	63	84
8	Accipitriformes	Pandionidae	*Pandionhaliaetus* (Linnaeus, 1758)	0	1
9	Apodiformes	Apodidae	*Apusnipalensis* (Hodgson, 1837)	20	25
10	Columbiformes	Columbidae	*Chalcophapsindica* (Linnaeus, 1758)	33	46
11	Columbiformes	Columbidae	*Columbapulchricollis* (Blyth, 1846)	0	3
12	Columbiformes	Columbidae	*Columbalivia* (J.F.Gmelin, 1789)	0	1
13	Columbiformes	Columbidae	*Spilopeliachinensis* (Scopoli, 1786)	82	48
14	Columbiformes	Columbidae	*Streptopeliaorientalis* (Latham, 1790)	23	20
15	Columbiformes	Columbidae	*Streptopeliatranquebarica* (Hermann, 1804)	9	1
16	Columbiformes	Columbidae	*Treronsieboldii* (Temminck, 1835)	14	43
17	Coraciiformes	Alcedinidae	*Alcedoatthis* (Linnaeus, 1758)	1	1
18	Cuculiformes	Cuculidae	*Cuculusoptatus* (Gould, 1845)	114	54
19	Cuculiformes	Cuculidae	*Cuculussparverioides* (Vigors, 1832)	0	3
20	Galliformes	Phasianidae	*Arborophilacrudigularis* (Swinhoe, 1864)	17	31
21	Galliformes	Phasianidae	*Bambusicolasonorivox* (Gould, 1863)	141	138
22	Galliformes	Phasianidae	*Lophuraswinhoii* (Gould, 1863)	0	3
23	Gruiformes	Rallidae	*Rallinaeurizonoides* (Lafresnaye, 1845)	1	0
24	Passeriformes	Aegithalidae	*Aegithalosconcinnus* (Gould, 1855)	1	1
25	Passeriformes	Campephagidae	*Pericrocotussolaris* (Blyth, 1846)	6	48
26	Passeriformes	Cettiidae	*Abroscopusalbogularis* (Moore, 1854)	87	143
27	Passeriformes	Cisticolidae	*Priniastriata* (Swinhoe, 1859)	12	2
28	Passeriformes	Cisticolidae	*Priniaflaviventris* (Delessert, 1840)	3	2
29	Passeriformes	Cisticolidae	*Priniainornata* (Sykes, 1832)	4	0
30	Passeriformes	Corvidae	*Corvusmacrorhynchos* (Wagler, 1827)	4	9
31	Passeriformes	Corvidae	*Dendrocittaformosae* (Swinhoe, 1863)	94	158
32	Passeriformes	Dicaeidae	*Dicaeumminullum* (Swinhoe, 1870)	6	44
33	Passeriformes	Dicaeidae	*Dicaeumignipectus* (Blyth, 1843)	3	3
34	Passeriformes	Dicruridae	*Dicrurusaeneus* (Vieillot, 1817)	47	104
35	Passeriformes	Dicruridae	*Dicrurusmacrocercus* (Vieillot, 1817)	13	4
36	Passeriformes	Estrildidae	*Lonchurastriata* (Linnaeus, 1766)	25	12
37	Passeriformes	Estrildidae	*Lonchurapunctulata* (Linnaeus, 1758)	4	1
38	Passeriformes	Fringillidae	*Pyrrhulanipalensis* (Hodgson, 1836)	0	1
39	Passeriformes	Hirundinidae	*Cecropisstriolata* (Schlegel, 1844)	18	8
40	Passeriformes	Hirundinidae	*Delichondasypus* (Bonaparte, 1850)	0	2
41	Passeriformes	Hirundinidae	*Hirundotahitica* (Gmelin, 1789)	32	30
42	Passeriformes	Hirundinidae	*Hirundorustica* (Linnaeus, 1758)	4	2
43	Passeriformes	Laniidae	*Laniuscristatus* (Linnaeus, 1758)	0	1
44	Passeriformes	Leiothrichidae	*Garrulaxcanorus* (Linnaeus, 1758)	4	5
45	Passeriformes	Leiothrichidae	*Garrulaxpoecilorhynchus* (Gould, 1863)	1	4
46	Passeriformes	Leiothrichidae	*Heterophasiaauricularis* (Swinhoe, 1864)	17	78
47	Passeriformes	Leiothrichidae	*Liocichlasteerii* (Swinhoe, 1877)	29	85
48	Passeriformes	Leiothrichidae	*Trochalopteronmorrisonianum* (Ogilvie-Grant, 1906)	0	1
49	Passeriformes	Monarchidae	*Hypothymisazurea* (Boddaert, 1783)	225	277
50	Passeriformes	Motacillidae	*Anthushodgsoni* Richmond, 1907	0	1
51	Passeriformes	Motacillidae	*Motacillaalba* (Linnaeus, 1758)	3	0
52	Passeriformes	Motacillidae	*Motacillacinerea* (Tunstall, 1771)	1	1
53	Passeriformes	Muscicapidae	*Copsychusmalabaricus* (Scopoli, 1786)	6	19
54	Passeriformes	Muscicapidae	*Myiomelaleucura* (Hodgson, 1845)	24	56
55	Passeriformes	Muscicapidae	*Myophonusinsularis* (Gould, 1863)	6	19
56	Passeriformes	Muscicapidae	*Niltavavivida* (Swinhoe, 1864)	1	1
57	Passeriformes	Oriolidae	*Oriolustraillii* (Vigors, 1832)	6	23
58	Passeriformes	Paridae	*Parusmonticolus* (Vigors, 1831)	6	8
59	Passeriformes	Passeridae	*Passermontanus* (Linnaeus, 1758)	36	0
60	Passeriformes	Pellorneidae	*Alcippemorrisonia* (Swinhoe, 1863)	397	472
61	Passeriformes	Pellorneidae	*Alcippebrunnea* (Gould, 1863)	296	432
62	Passeriformes	Phylloscopidae	*Phylloscopusinornatus* (Blyth, 1842)	0	6
63	Passeriformes	Phylloscopidae	*Phylloscopusborealis* (J.H.Blasius, 1858)	0	1
64	Passeriformes	Pittidae	*Pittanympha* Temminck & Schlegel, 1850	0	2
65	Passeriformes	Pycnonotidae	*Hypsipetesleucocephalus* (Gmelin, 1789)	496	388
66	Passeriformes	Pycnonotidae	*Pycnonotussinensis* (Gmelin, 1789)	456	217
67	Passeriformes	Pycnonotidae	*Spizixossemitorques* (Swinhoe, 1861)	29	41
68	Passeriformes	Sturnidae	*Acridotheresjavanicus* (Cabanis, 1851)	26	0
69	Passeriformes	Sturnidae	*Acridotherescristatellus* (Linnaeus, 1758)	2	0
70	Passeriformes	Timaliidae	*Pomatorhinusmusicus* (Swinhoe, 1859)	285	446
71	Passeriformes	Timaliidae	*Pomatorhinuserythrocnemis* (Gould, 1863)	127	199
72	Passeriformes	Timaliidae	*Stachyridopsisruficeps* (Blyth, 1847)	461	423
73	Passeriformes	Turdidae	*Turduschrysolaus* (Temminck, 1832)	2	6
74	Passeriformes	Turdidae	*Turduspallidus* (Gmelin, 1789)	1	1
75	Passeriformes	Vireonidae	*Erporniszantholeuca* (Blyth, 1844)	22	52
76	Passeriformes	Zosteropidae	*Yuhinabrunneiceps* (Ogilvie-Grant, 1906)	2	9
77	Passeriformes	Zosteropidae	*Zosteropssimplex* (Swinhoe, 1861)	248	65
78	Pelecaniformes	Ardeidae	*Bubulcusibis* (Linnaeus, 1758)	7	8
79	Pelecaniformes	Ardeidae	*Egrettagarzetta* (Linnaeus, 1766)	6	7
80	Pelecaniformes	Ardeidae	*Gorsachiusmelanolophus* (Raffles, 1822)	3	3
81	Pelecaniformes	Ardeidae	*Nycticoraxnycticorax* (Linnaeus, 1758)	0	2
82	Pelecaniformes	Ardeidae	*Ardeaalba* (Linnaeus, 1758)	1	0
83	Piciformes	Megalaimidae	*Psilopogonnuchalis* (Gould, 1863)	538	567
84	Piciformes	Picidae	*Yungipicuscanicapillus* (Blyth, 1845)	37	46
85	Strigiformes	Strigidae	*Glaucidiumbrodiei* (Burton, 1836)	1	9
